# Effect of Melt-Compounding Protocol on Self-Aggregation and Percolation in a Ternary Composite

**DOI:** 10.3390/polym12123041

**Published:** 2020-12-18

**Authors:** Ji Hwan Kim, Joung Sook Hong, Akira Ishigami, Takashi Kurose, Hiroshi Ito, Kyung Hyun Ahn

**Affiliations:** 1 School of Chemical and Biological Engineering, Institute of Chemical Processes, Seoul National University, Seoul 08826, Korea; realzh91@snu.ac.kr (J.H.K.); ahnnet@snu.ac.kr (K.H.A.); 2 Research Center for GREEN Materials & Advanced Processing, Graduate School of Organic Materials Science, Yamagata University, Yamagata 992-8510, Japan; Akira.Ishigami@yz.yamagata-u.ac.jp (A.I.); takashi.kurose@yz.yamagata-u.ac.jp (T.K.); ihiroshi@yz.yamagata-u.ac.jp (H.I.)

**Keywords:** biopolymer blend, carbon black, particle aggregation, conductivity

## Abstract

A ternary composite of poly(lactic acid) (PLA), poly(caprolactone) (PCL), and carbon black (CB) shows the PCL-induced CB self-aggregation and percolation formation when the amount of the PCL phase as the secondary phase is as small as the amount of CB. Furthermore, when the drop size of the PCL phase becomes smaller, the ternary composite forms a percolation of high order structure, resulting in a remarkable enhancement of the electrical conductivity (~4 × 10^−2^ S/m with 4 wt.% CB). To further control the percolation structure, the composite fabrication is controlled by splitting a typical single-step mixing process into two steps, focusing on the dispersion of the secondary PCL phase and the CB particles separately. Under the single-step mixing protocol, the ternary composite shows a structure with greater CB aggregation in the form of a high aspect ratio and large aggregates (aggregate perimeter~aggregate size 0.7). Meanwhile, the two-step mixing process causes the CB aggregates to expand and create a higher structure (aggregate perimeter~aggregate size 0.8). The reduced size of the secondary phase under a mixing condition with high shear force prior to the addition of CB provides a larger interfacial area for CB to diffuse into the PCL phase during the subsequent mixing step, resulting in a further expansion of CB aggregation throughout the composite. The particle percolation of such a high order structure is attributed to high storage modulus (G′), high Young’s modulus, high dielectric loss (ε″), and negative–positive switching of dielectric constant at high frequency (of 103 Hz) of composite.

## 1. Introduction

A conductive biopolymer composite (henceforth CBC) is a composite containing a mixture of biopolymers with a type of conductive particle, such as carbon black, metal powder, carbon nanotube, graphene, or a combination of these, with considerable potential in industrial applications of high value-added products such as textiles and biomedical sensors [1,2,3,4,5]. The compounding process is the most economical method to produce CBC. One of the key challenges in the CBC fabrication is to realize conductivity without sacrificing its mechanical performance and processability, because it is difficult to realize a synergistic effect between tensile mechanical performance and the functionality when large amounts of particles are used. Thus, one of the major goals for successful CBC fabrication is to induce an electrical percolation threshold at low particle concentration as a critical amount of particles to form a continuous conductive network.

Among the diverse conductive particles, carbon black (CB) has been widely used to induce electrical performance due to its low cost and good dispersibility. The percolation of CB in a polymer depends significantly on the surface properties of CB, as well as on the interaction between the CB particles and the polymer matrix [6,7]. The composites often show a significant reduction in the percolation threshold when manipulating the particle distribution in an immiscible blend system [8,9,10,11,12,13]. This is realized based on selective localization of CB particles in a multi-phase system due to a thermodynamic reason [14,15]. When CB is incorporated into immiscible phases, it becomes localized in one specific phase or at the interface between the phases according to the balance of the spreading coefficient of the particles between the phases [7,8,9,10,11,12,13,14,15]. Specifically, if CB is densely dispersed in one phase or at the interface of the blend and it eventually shows a co-continuous structure, the composite can form a double-percolated structure, giving rise to an increase in the electrical conductivity, even with a lower particle concentration compared to those of typical binary composites [8,9,10,11,12,13]. For example, with polyethylene (PE)/polystyrene (PS) 45/55 blend [13], the composite shows a remarkable reduction in the percolation threshold depending on where the CB is localized in the system. When CB particles are selectively localized only in PE phase and the CB/PE/PS forms a co-continuous structure, the percolation threshold is obtained with an addition of 3% CB. However, the addition of 0.4% CB induces the percolation threshold if the CB particles are localized only at the interface between PE and PS.

It remains challenging to maintain the percolation structure to the product stage when dealing with the instability of the co-continuous structure. Nonetheless, the selective localization of particles in ternary composite is promising for the effective design of CBC. Recently, the addition of a secondary phase with higher affinity to particles was found to reduce the percolation threshold, especially when the concentration of secondary phase is comparable to or less than the particle concentration [16,17]. Localization of CB in a multi-phase system reduces the percolation threshold less than that of a binary composite, with an electrical percolation threshold typically in the range of 3–5 wt.% CB. The percolation threshold of polycarbonate/CB composites can be reduced from 12 to 5 wt.% after the addition of 3 wt.% polyamide 6, which has high affinity to CB [16]. For poly(lactic acid) (PLA), it is reduced from 3.7 to 2.9 wt.% after the addition of 4 wt.% poly(caprolactone) (PCL) [17]. Despite the reduced electrical threshold, it is generally in the electrostatic–antistatic conductivity level. Extra CB should be incorporated into composites beyond the percolation threshold so that the conductivity level exceeds 10^−4^ S/m. In this case, the composite still requires a large number of particles to achieve the target electrical conductivity, which deteriorates tensile mechanical properties of the CBC. Therefore, for successful CBC application, a deeper understanding of the particle aggregation and percolation formation in a ternary system is necessary so that the percolation threshold ultimately can be reduced while increasing the conductivity with a low content of CB. Specifically, for PLA, one of the most promising biopolymers, it is crucial to develop a technology that realizes CBC with a low concentration of particles, to guarantee a positive synergistic effect over the inherited brittleness of PLA (elongation at break <20%) [18,19].

According to the earlier work on PLA-based ternary composites [17], the dispersion of CB in the PLA matrix is significantly affected by the secondary PCL phase. For the PLA/PCL blend, the selective localization of CB in the PCL phase has a promising effect, with a decrease in percolation threshold [17]. Double percolation (50 wt.% PCL/PLA blend with 4 wt.% CB) weakly improves the electrical performance (3 × 10^−9^ S/m). When the concentration of PCL is reduced to less than 6 wt.% in 4 wt.% CB/PLA composite, the CB particles aggregate in the vicinity of the high-affinity PCL phase, and the electrical conductivity increases from 10^−7^ (PLA/CB composite) to 10^−3^ S/m (PLA/PCL/CB composite). A small amount of PCL induces the aggregation of CB and provides a route to design CBC other than by selective localization. From this perspective, the dispersion of the PCL phase itself can be another important factor to consider when controlling the dispersion of CB particles in a ternary composite. Thus, for a more in-depth understanding how the secondary phase (PCL) induces particle aggregation and percolation in a ternary composite, this study focuses on the change of percolation structure in ternary composites depending on the mixing protocol used. Specifically, under the assumption that the particle structure in a ternary composite system (PLA/PCL/CB) is significantly affected by competitive dispersion between the particles and the secondary phase during the mixing process, the ternary composites tested here undergo several mixing processes differing in terms of the mixing conditions and sequence. For this purpose, a different mixing process is employed, with two batch-type mixers having different screw configuration and rotation speed. In this study, a typical internal batch mixer and a high-shear kneading mixer are used [20]. The mixing sequence is designed to distinguish the blend morphology evolution from the particle dispersion during the mixing process. The particle aggregates are characterized by morphology analysis to compare the structural changes of the composites.

## 2. Experimental

### 2.1. Materials

PLA (4032D, ${\bar{M}}_{w}$ = 181 kg/mol, ${\bar{M}}_{w}$/${\bar{M}}_{n}$ = 2.01, ρ = 1.24 g/cm^3^) was purchased from NatureWorks (Minnetonka, MN, USA), PCL (CAPA^®^ 6800, Mw = 80 kg/mol, MFI (160 °C, 2.16 kg) = 0.24 g/min, ρ = 1.1 g/cm^3^) from Perstorp (Malmö, Sweden), and carbon black (CB) (xc-72r, mean particle diameter = 30 nm, ρ = 1.92 g/cm^3^) from Cabot Corp (Boston, MA, USA).

### 2.2. Sample Preparation

Before preparing the samples, the materials were dried in a vacuum oven overnight at 80 °C for PLA and CB, and at 40 °C for PCL. The composite samples were prepared by a melt-compounding process at 180 °C (the viscosity ratio of PCL to PLA is 0.83 at this temperature). Two different batch-type mixers were employed, an internal batch mixer (B) and a high-shear kneading mixer (H). A rotor-mixer (Rheocomp^®^ 600, MKE, Daejeon, Korea) was used as an internal batch mixer (B), and a high-shear processing machine (NHS 2-28; Niigata Machine Techno Co., Ltd., Niigata, Japan [20]) was used as the high-shear kneading mixer (H). The high-shear processing machine is composed of two sections; a plasticization–injection section that plasticizes the resin and injects an arbitrary amount, and a high-shear kneading section. Details of the high-shear kneading mixer are described elsewhere [20].

The composition of the composite is presented in Table 1. The nomenclature for the ternary composites is α_PCLβCBγ, where α refers to the mixing method (B, H, or both), and β and γ correspondingly denote the weight percentage of PCL and CB based on the PLA matrix. As an example, for B_PCL4CB4, the PLA matrix was mixed with 4 wt.% PCL and 4 wt.% CB through a single-step mixing in the internal batch mixer (B). In this study, PCL4CB4 composites were fabricated by mixing methods with different mixers (B, H) and with different mixing sequences (B, H, BB, HB) (Table 2). For B_PCL4CB4, dry-mixed PLA, PCL, and CB were loaded slowly into the internal batch mixer (B) at 180 °C for 2 min. Subsequently, melt-compounding was carried out for 6 min at a screw speed of 100 rpm. The BB_PCL4CB4 composite was prepared by a two-step mixing process. In the first step, dry-mixed PLA and PCL were pre-melted and melt-mixed under 180 °C for 6 min at 100 rpm using the internal batch mixer (B). Secondly, CB powder was added into the PLA/PCL blend, and was mixed with an identical mixing condition for 6 min. Here, BB indicates that the minor PCL phase was dispersed within the PLA matrix first by the batch mixer (B_PCL4), after which the blend was mixed with CB using the same mixer in the second step (BB_PCL4CB4). For the H_PCL4CB4 composite, dry-mixed PLA, PCL, and CB were loaded into the mixer and melt-mixed in one step using the high-shear processing machine at 180 °C for 30 s with a screw speed of 1000 rpm (apparent shear rate of 3000 s^−1^, approximately). Regarding the HB_PCL4CB4 composite, the PCL/PLA blend was prepared with the high-shear kneading mixer (H) first, with this sample referred to as H_PCL4. The H_PCL4 was then pelletized using a pelletizer (Ciran Cutter, Harmo Co., Ltd., Nagano, Japan). In the second step, H_PCL4 was mixed with 4 wt.% CB using the internal batch mixer (B) under a condition identical to that used to devise the B_PCL4CB4 sample. After compounding, the samples were compression-molded into a disk with a thickness of 0.45 mm and a diameter of 25 mm using a hot press (CH4386, Carver Inc., Wabash, IN, USA) at 180 °C for 6 min for characterization.

### 2.3. Characterization

The complex dielectric permittivity ($\varepsilon^{*}\left(\omega \right)=\;\varepsilon \;\prime \left(\omega \right)+j\;\varepsilon \;\prime \prime \left(\omega \right),\;j=\sqrt{-1}$) was measured at room temperature using an impedance/gain-phase analyzer (SI 1260, AMETEK^®^, Berwyn, PA, USA) with a custom-made two-point probe instrument. Contact resistance was minimized by setting the electrodes of the two-point probe geometry to a needle-like shape, with both sides of the disk-shaped specimens also coated with silver paste to measure the volume resistivity across the entire area of the specimen. The tests were conducted in a frequency range of 10 to 10^6^ Hz, and the DC conductivity (σ_dc_) was evaluated using the dielectric loss  ε ″(ω) data at low frequency ($\sigma_{dc}=\varepsilon_{0}{\left[\omega \;\varepsilon \;\prime \prime \left(\omega \right)\right]}_{\omega \to 0},$ where $\varepsilon_{0}$ is the vacuum permittivity$\;(\varepsilon_{0}=8.854\times 10^{-12}$ F/m)).

For rheological characterization, linear viscoelastic measurements were carried out at 180 °C using a strain-controlled rheometer (RMS800, Rheometrics, Piscataway, NJ, USA) with a 25 mm parallel plate fixture. For neat PLA, a dynamic time sweep test at 180 °C for 20 min was carried out at a frequency of 1 rad/s in order to ensure thermal stability during the rheological test, indicating no detectable change in the modulus. Dynamic strain sweep tests were performed at a fixed frequency, 1 rad/s, to determine the linear viscoelastic regions, after which the dynamic storage moduli (G′(ω)), loss moduli (G″(ω)), and complex viscosity (η*(ω)) were determined through dynamic frequency sweep tests ranging from 0.1 to 100 rad/s at a fixed strain level within the linear viscoelastic region.

The morphology of the composite was observed through field-emission scanning electron microscopy (FE-SEM, SUPRA-55VP, Carl Zeiss, Oberkochen, Germany) at an operating voltage of 2 kV. For the observation, the surface of the specimen was prepared by fracturing after quenching with liquid nitrogen (cryo-fracturing method) and the obtained surface was sputtered with a layer of platinum. The specimens for TEM imaging were prepared using a cryo-ultramicrotome (EM UC6, LEICA, Wetzlar, Germany) with a diamond knife.

**Figure 1:**
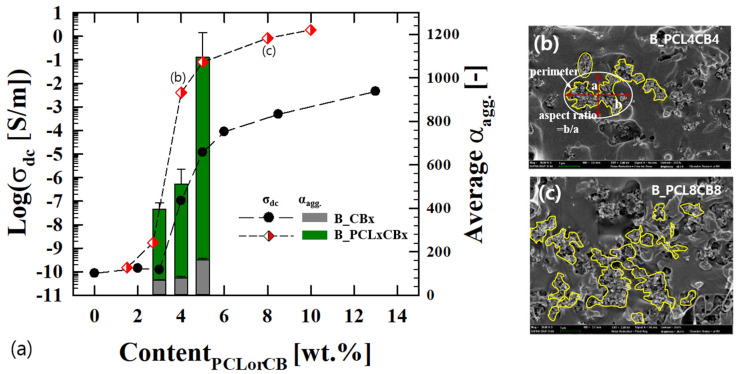
(**a**) DC conductivity (σ_dc_) and average aggregate size (α_agg._) of B_CB*x* binary and B_PCL*x*CB*x* ternary composites depending on the PCL or CB content. As indicated in (**b**), each aggregate dispersed in the composite was selected and examined to characterize the aggregate size. (**c**) B_PCL8CB8 was difficult to characterize due to uncertain boundaries of the aggregates and the expanded percolated structure.

2D SEM images were analyzed for quantitative characterization of the composite morphology. For each composite, more than 100 CB aggregates from SEM images were selected (as indicated in Figure 1) and analyzed using the image analysis software Image J (National Institutes of Health, Bethesda, MD, USA). For individual CB aggregates, the number of CB particles constituting each aggregate (aggregate size, α_agg_ = area of the aggregate/area of a single particle), the perimeter indicating the outline of the aggregate, and the aspect ratio of a circle embracing the aggregate were measured. The aggregate size, perimeter, and aspect ratio were number averaged (=$\sum n_{i}x_{agg,\;i}/\sum n_{i})$, where *n_i_* is the number of aggregates) and weight-averaged $\left(\sum n_{i}x_{agg,i}^{2}/\sum n_{i}x_{agg,i}\right)$ to represent the geometric characteristics of the composites. The perimeter informs us of how irregular the aggregates expand. If the composites having the same average α_agg_ value (α_agg. avg_) show different perimeters, the composite with a larger perimeter will have the structure with a more irregular shape.

## 3. Results and Discussion

When CB particles are added to an immiscible blend, they are distributed in different proportions between the phases depending on the balance of the chemical affinity of the particles between the phases and on the mixing history [21,22,23,24]. If a particle has higher affinity to the minor phase and the concentration of the minor phase is as low as the particle content, the particles accumulate densely in the minor phase. Despite the fact that the particles form a percolation structure inside the minor phase, the composite does not realize the electrical conductivity due to the disconnections of the minor phase. Thus, the dispersion and distribution of the minor phase affects the particle dispersion and microstructure formation. In this study, a ternary composite system (PLA/PCL/CB) is investigated to understand how a secondary PCL phase with higher affinity to CB than PLA affects the particle dispersion and electrical conductivity of the composite with a small amount of PCL (Section 3.1). For a ternary composite with a fixed concentration of CB and PCL, the effect of mixing method on the particle aggregation is investigated based on morphology analysis (Section 3.2). The particle structure of the ternary composite is characterized based on an electrical and rheological analysis (Section 3.3).

### 3.1. PCL-Induced CB Self-Aggregation

PLA. PLA/CB binary composites display electrical conductivity when the amount of CB exceeds a certain threshold. Figure 1a presents the DC conductivity (σ_dc_) of the composites depending on the concentration of CB (ϕ_CB_), from 0 to 13 wt.%. The σ_dc_ of binary composite shows a scaling behavior depending on the CB content when it exceeds an electrical threshold of 3.73% CB (σ_dc_~ϕ_CB_^2.98^ [17]). When a certain amount of the secondary PCL phase (4 wt.%), which has higher chemical affinity to particles than the PLA matrix, is added to these binary composites, the ternary composite of PLA/PCL/CB shows different electrical performance depending on the CB content (σ_dc_~ϕ_CB_^2.44^ with the addition of 4 wt.% PCL [17]). Additionally, the composite shows a significant increase in the σ_dc_ value. The σ_dc_ of the 4 wt.% CB/PLA composite increases from 1 × 10^−7^ S/m to 4 × 10^−3^ S/m with 4 wt.% PCL. In addition to the effect of a small amount of the secondary PCL phase on the electrical performance which outruns that of the binary composites, when a composite contains CB and PCL at an equivalent mixing ratio, it shows a pronounced increase in the electrical conductivity (σ_dc_~ϕ_CB_^3.96^), as shown in Figure 1a. Figure 1a shows the σ_dc_ of ternary composites (B_PCLxCBx) depending on the PCL or CB content. The σ_dc_ of the ternary composites increases by approximately four orders of magnitude compared to the binary composites above the electrical threshold. This outcome is attributed to PCL-induced self-aggregation of CB particles. As shown in Figure 1a, the average aggregate size (α_agg._) of the ternary composite is significantly increased with the increase in CB content compared to that of the binary composite. When ϕ_CB_ increases from 3 to 5 wt.%, α_agg_ of the ternary composite increases from 400 to 1100 (for binary composite, from 68 to 160) (Table 3). Interestingly, the perimeter and aspect ratio of the aggregates also increase rapidly (Table 3), meaning that the CB aggregates become more irregular in shape and extend around neighboring aggregates to form percolation. As shown in Figure 1b, the composite with 4 wt.% CB and 4 wt.% PCL shows that the aggregates with irregular shapes are gathered to form a temporary network. Above 4 wt.% CB, the aggregate size exceeds a few micrometers, forming a network of the aggregates. For B_PCL8CB8 (Figure 1c), the aggregates are densely agglomerated, and it is even difficult to select individual aggregates. Above 4 wt.% CB, the concentration of CB becomes saturated, forming a percolation structure with high σ_dc_. To discriminate the effect of PCL-induced CB aggregation on percolation formation clearly, the concentration of the model composite is fixed at 4 wt.% CB and PCL.

Because the percolation of PLA/PCL/CB composite strongly depends on the concentration of the secondary phase (PCL) and its ratio with CB, the morphology control of the PCL phase is important not only in inducing particle aggregation, but also in forming a network of aggregates with only a small number of particles. The next section investigates whether the change in dispersion and size of the secondary PCL phase can lead to changes in particle aggregation and percolation.

### 3.2. Mixing Effect on PCL-Induced CB Self-Aggregation

During the mixing process of the ternary composite, mechanical shear force critically affects the dispersion of CB and the secondary phase. In this complicated process, CB particles diffuse into the secondary PCL phase due to its chemical preference to PCL relative to PLA. It can be assumed that the CB particles will have more chances to diffuse into the PCL phase, leading to a homogeneous distribution of CB in the PCL domain as the size of the PCL phase is reduced, and the formation of connections between the CB aggregates can be realized as the CB content further increases. In addition, the effect of mixing conditions on CB aggregate formation in a ternary composite with a fixed composition (PLA/PCL4/CB4) is tested under diverse mixing processes with different shearing force levels. In this study, two batch mixers with different designs (an internal batch mixer (B) and a high-shear kneading mixer (H)) are employed to fabricate the composites through single-step or two-step mixing processes (Table 2).

**Figure 2:**
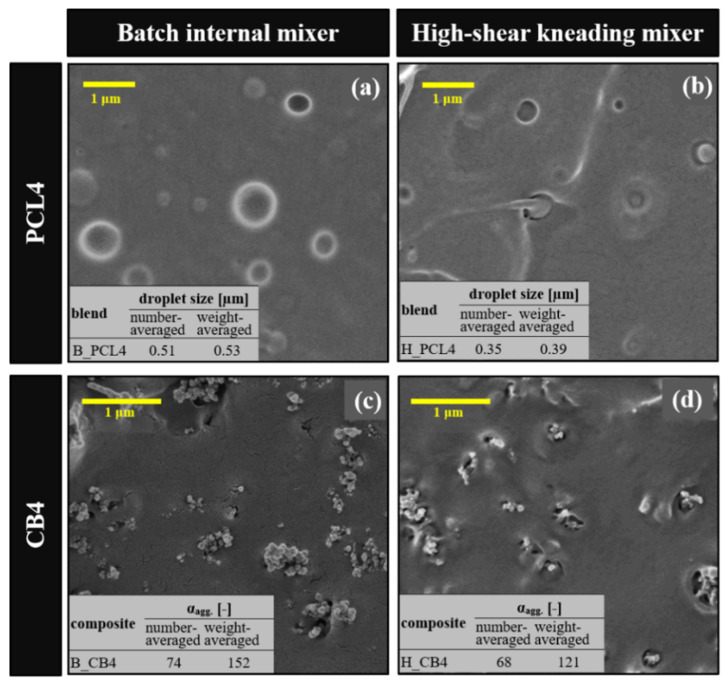
SEM images of the fractured surface of (**a**,**b**) PLA/PCL blends (PCL4) and (**c**,**d**) 4 wt.% CB/PLA binary composites (CB4) prepared by batch mixing and high-shear kneading processing.

Before the ternary composite is studied with these mixers, the dispersion of the PCL phase or CB particles is investigated to determine different dispersion characteristics of the mixer. Figure 2 compares SEM images of the binary composites (4 wt.% CB) and the PLA/PCL blends (4 wt.% PCL) fabricated by the internal batch mixer (B_CB4 and B_PCL4) and by the high-shear kneading process machine (H_CB4 and H_PCL4). The number-averaged drop size of B_PCL4 (~0.51 µm) is larger than that of H_PCL4 (~0.35 µm) (Figure 2 inset). In addition, the number-averaged size of the particle aggregate of B_CB4 (~74) is larger than that of H_CB4 (~68) (Figure 2 inset). As expected, the high-shear force mixing of the PCL/PLA leads to a blend with a smaller drop size due to dominant breakup behavior against coalescence under high shear.

The composite also shows a distribution of smaller aggregates even with a short mixing time under high-shear mixing, resulting in an insulation conductivity level (~10^−10^ S/m for H_CB4, while it is 10^−7^ S/m for B_CB4). Despite the decreased aggregate size, the particle aggregates are not close enough to contact with neighboring aggregates. The results of this test (Figure 2) demonstrate that the dispersion of CB particles (or the secondary PCL phase) can be affected by the mixing method. A simple homogeneous dispersion of small aggregates is not desirable for CBC fabrication, as previous studies indicate that less mixing is required to obtain a composite with high aggregate structure [21,22,23]. The mixing procedures for conductive composites must be carefully controlled to avoid excessive dispersion of the CB particles and a lack of dispersion of the secondary phase.

**Figure 3:**
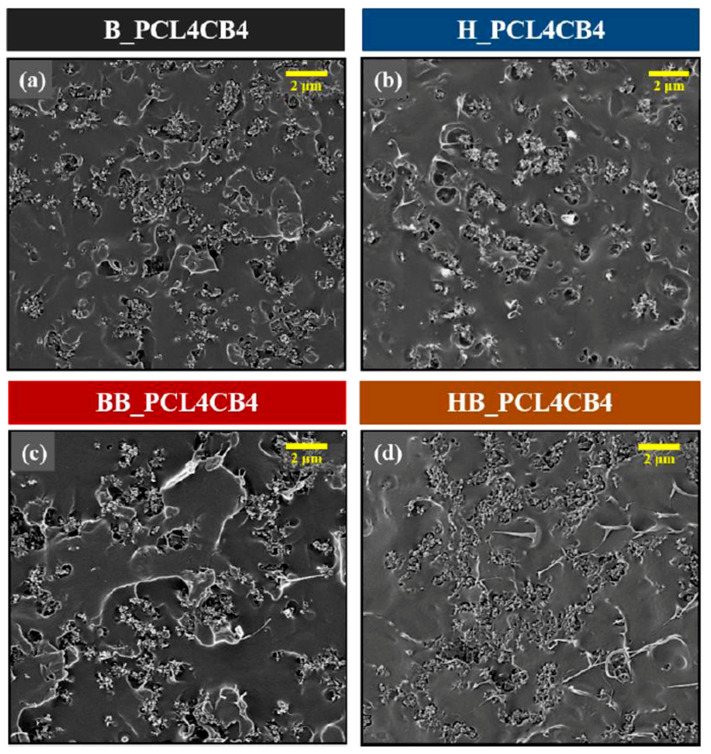
SEM images of the fractured surface of the ternary composite (PCL4CB4) depending on the mixing method: (**a**) B_PCL4CB4, (**b**) H_PCL4CB4, (**c**) BB_PCL4CB4, and (**d**) HB_PCL4CB4.

**Figure 4:**
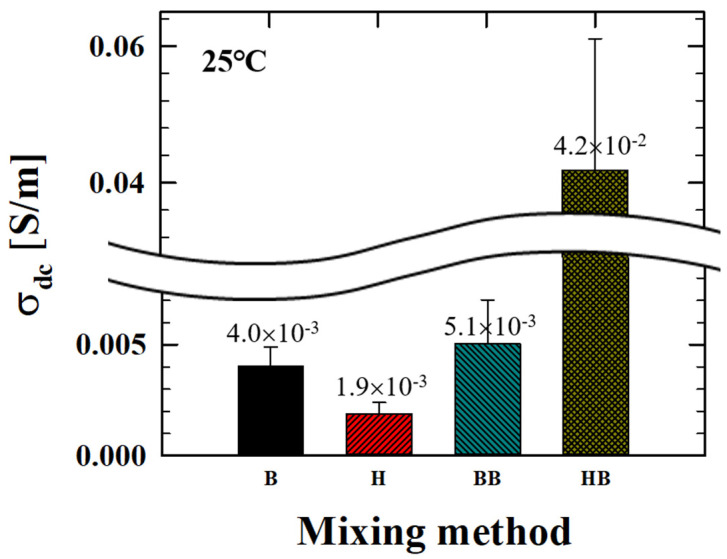
Electrical conductivity (σ_dc_) of a ternary composite depending on the mixing method.

Ternary composites containing fixed content of CB and PCL (4PCL and 4CB) are produced with different mixers and mixing sequences. Figure 3 shows SEM images of these ternary composites depending on the mixing method, and the corresponding σ_dc_ are compared in Figure 4. Regardless of the mixing method used, the addition of the secondary PCL phase (4 wt.%) remarkably induces CB aggregation compared to H_CB4 or B_CB4 (Figure 2). In addition to the PCL-induced CB aggregation, the ternary composites have CB aggregates of noticeably different structures depending on the mixing method. As observed in Figure 2, B_PCL4CB4 fabricated at a lower shear mixing condition shows more particle aggregation than H_PCL4CB4 (Figure 3a,b). As for mixing sequence, CB aggregates in ternary composites fabricated via single-step mixing are more scattered and show more disconnections between the aggregates compared to the composite via the two-step mixing process. During two-step mixing, both PCL and CB are mixed sequentially under the assumption that a homogeneous distribution of the secondary PCL phase with a small size will enhance the distribution of the CB particles. The dispersion of the PCL phase occurs in the first step (B_PCL4 or H_PCL4), after which CB is added to the blend using the same mixer (B). Two-step mixing forms CB aggregates with more contact with neighboring aggregates to form a network and better electrical performance. The σ_dc_ of H_PCL4CB4 is significantly increased to 1.9 × 10^−3^ S/m from the insulation level of H_CB4. B_PCL4CB4 has a higher σ_dc_ (~4 × 10^−3^ S/m) than H_PCL4CB4 (Figure 4). Moreover, the σ_dc_ of BB_ and HB_PCL4CB4 is higher than that of B_ or H_PCL4CB4. Among them, HB_PCL4CB4 has a much higher σ_dc_ than BB_PCL4CB4, which is attributed to a remarkable distribution of CB aggregates throughout the composite as can be seen in Figure 3d.

**Figure 5:**
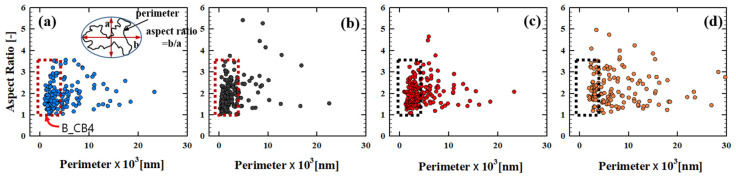
Aspect ratio (AR) vs. perimeter (P) of the PCL4CB4 ternary composites: (**a**) H_PCL4CB4, (**b**) B_PCL4CB4, (**c**) BB_PCL4CB4, and (**d**) HB_PCL4CB4 (the box with dotted line represents the distribution of AR, perimeter of aggregates in B_CB4).

In order to distinguish structural difference depending on the mixing method in more detail, a quantitative morphology analysis is performed with 2D SEM images. From each 2D image, CB aggregates are discriminated, as demonstrated in the inset of Figure 5a, and analyzed to define the geometric characteristics of the CB aggregates—size, perimeter, and aspect ratio. Figure 5 presents the outcome of the image analysis of the ternary composites depending on the mixing method, as summarized in Table 4. Compared to the average aggregate size (α_agg_) of B_CB4 (74) or H_CB4 (68) in Figure 2, the addition of the secondary PCL phase pronouncedly increases α_agg._ of the ternary composite, i.e., 510 and 470 for the B_ and H_PCL4CB4 composites, respectively (Table 4). When CB aggregates distributed in B_ and H_PCL4CB4 composites are compared with the distribution of CB aggregates in B_CB4 binary composite, most aggregates are extended and form an irregular shape, as shown in Figure 5. As the point representing each CB aggregate is scattered outside of the square, it can be understood that the secondary PCL phase induces the aggregation of CB with a larger perimeter or a higher aspect ratio compared to the B_CB4 outcome. In addition, the secondary PCL phase enhances the variation of CB aggregation further depending on the mixing method. For H_PCL4CB4 (Figure 5a), CB aggregates have a lower aspect ratio than B or BB_ PCL4CB4 (Figure 5b,c), and the distribution of CB aggregates becomes narrower than other composites. B_ PCL4CB4 (~aspect ratio of 2.1 and perimeter of 3980) has more CB aggregates with a higher structure compared to H_ PCL4CB4 (~aspect ratio of 1.9 and perimeter of 3830) (Table 4). This indicates that single-step mixing with a high-shear kneading process is too intensive to control the aggregation of CB particles and to control the dispersion of the PCL phase at the same time. Less shear force is desirable to induce particle aggregation. Compared to the ternary composite from a single-step mixing, two-step mixing expands the distribution of the CB aggregates to the region with a higher perimeter and higher aspect ratio (Figure 5c,d). Especially for HB_PCL4CB4, many CB aggregates are distributed in the higher perimeter and higher aspect ratio region compared to the other ternary composite (PCL4CB4) (Figure 5d). The average perimeter of CB aggregates in HB_ PCL4CB4 (~7720) is noticeably larger than that of BB_ PCL4CB4 (~4800) despite the fact that the aspect ratio of both composites is similar (2.2 and 2.2 for BB_ and HB_ PCL4CB4, respectively, Table 4), indicating that CB aggregates are highly extended throughout the composite to form percolation.

**Figure 6:**
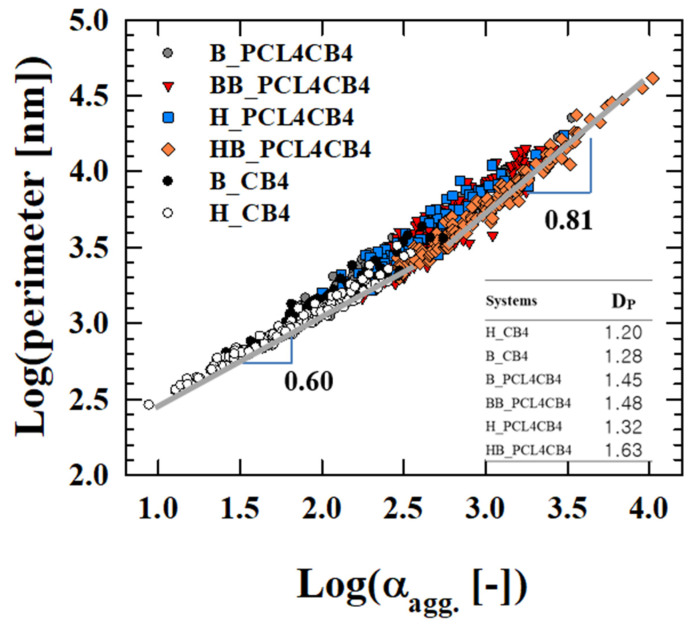
Plots of perimeter vs. α_agg._ of CB aggregates in binary and ternary composites depending on the mixing method. The inset table lists the perimeter-based fractal dimension (D_p_) for the composites. As D_p_ deviates from 1.0, the CB aggregates show a more irregular shape, deviating from spherical and show a branched structure if it exceeds 1.32 [26].

The perimeter and aggregate size of all CB aggregates dispersed in four composites are plotted in a single graph in Figure 6, which shows a scaling relationship (P~ADp2, where P and A are the perimeter and particle aggregate size respectively, and D_p_ is the fractal dimension [25]) (Figure 6). The CB aggregates in the binary composites (B_CB4, H_CB4) set into a master plot having a relationship of $P\sim A^{0.6}$ (D_p_/2 = 0.6). The scaling parameter increases for the ternary composites to $P\sim A^{0.8}$ (D_p_/2 = 0.8). As D_p_ deviates from 1.0, the CB aggregates show a more irregular shape, deviating from spherical. According to earlier work [26], D_p_ is 1.15 for ellipsoidal aggregates, and it increases to 1.29 for linearly extended aggregates. It exceeds 1.32 for aggregates with a branched structure. As shown in Figure 6, CB aggregates for the binary composites increase irregularity from ellipsoidal to a slender form [26]. For ternary composites, CB aggregates become more irregular, forming higher-order structures. HB_PCL4CB4 has a high value of D_p_ (~1.63) compared to B_ and BB_composites, resulting in a percolation structure with higher connectivity and leading to higher electrical conductivity than in other ternary composites (Figure 4). Meanwhile, the H_composite has a low value of D_p_ (~1.32), indicative of a percolation structure with low connectivity and lower electrical conductivity.

These results indicate that the formation of a percolation structure with high connectivity is caused by a homogeneous dispersion of the secondary PCL phase with a small drop size through high-shear kneading before the addition of CB particles. As the drop size is smaller, the dispersion of the PCL phase enhances the contact of PCL with the CB particles and builds up a network between them. This suggests that decoupled mixing of a secondary phase with stronger affinity to particles from particle dispersion through a sequential mixing process provides a significant possibility to control the particle dispersion for percolation formation as compared to that by single-step mixing.

### 3.3. Dielectric and Rheological Characterization of the Ternary Composites

The morphological analysis of CB aggregates shows that the ternary composites with identical contents of CB and the secondary phase can have different CB aggregation and percolation structure depending on the mixing method. When the secondary PCL phase has a smaller drop size prior to the addition of CB particles, CB aggregates expand to form a percolation structure throughout the ternary composite (Figure 6 and Table 4). An analysis of the bulk electrical and rheological properties of the composites provides more details on the three-dimensional percolation structure.

**Figure 7:**
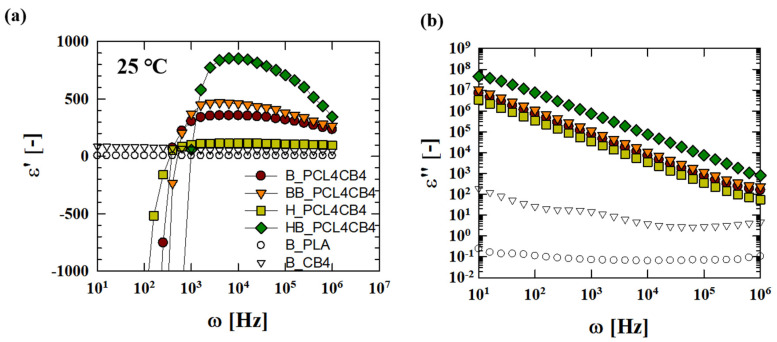
Dielectric properties of ternary composites depending on the mixing method: (**a**) Dielectric constant (ε′) and (**b**) dielectric loss (ε″).

Figure 7 depicts the dielectric response of the ternary composites as a function of frequency. The addition of 4 wt.% CB particles to the PLA matrix increases the dielectric constant (ε’) over the entire frequency region, and the binary composite shows a frequency-dependent dielectric constant. The dielectric constant of neat PLA is 6.6 at 10 kHz, whereas the dielectric constant of the B_CB4 composite is as high as 52.4 at the same frequency. The increase in the dielectric constant in the composite is mainly attributed to the action of the CB particles dispersed throughout the matrix as a nano-capacitor and to the space charges surrounding the interfaces between the particles and polymer matrix, giving rise to enhanced interfacial polarization [2,27,28,29]. Then, if the particle content is fixed, the dielectric constant of the composite increases as the interfacial area between the matrix and particle increases. For the ternary composites, the dielectric constant increases to the levels as high as 356, 460, 116, and 852 at 10 kHz for B, BB, H, and HB_PCL4CB4 composites, respectively. It indicates that the ternary composites have CB aggregates with a higher interfacial area. These aggregates play a role as a micro-capacitor in the composite. For the ternary composites, the interfacial area of the micro-capacitor increases depending on mixing method in the sequence of HB≫BB≥B≥H, which is the same sequence with the aggregate size identified earlier in the image analysis (Table 4).

In addition, the dielectric constant of the ternary composites shows a large negative value in the low frequency region, and then changes from negative to positive as the frequency increases. A negative dielectric constant indicates the formation of a continuous conducting pathway in the composite via direct contact between the conductive particles [30]. The switching frequency, at which the dielectric constant changes sign, is strongly related to the formation of a three-dimensional structure of the particles in the polymer matrix [31]. Network structure formation on a larger scale would lead to a higher switching frequency. The switching frequency of the ternary composites increases in the sequence of HB (~1000 Hz) > BB (~630 Hz) > B = H (~400 Hz) (Figure 7a). Correspondingly, the ternary composites show an increase in the dielectric loss (ε”) that is several orders of magnitude higher than that of B_CB4 (Figure 7b). For the ternary composites, the dielectric loss is proportional to ω^−1^ over the frequency and exhibits DC conductance. This arises because the CB aggregates act as a conducting pathway, inducing a leakage current throughout the composite. In this regard, HB_PCL4CB4 composite can be expected to have a three-dimensional conducting network structure of CB particles with stronger connectivity throughout the composite, leading to the highest value of the dielectric loss as well as the higher switching frequency compared to the other ternary composites.

**Figure 8:**
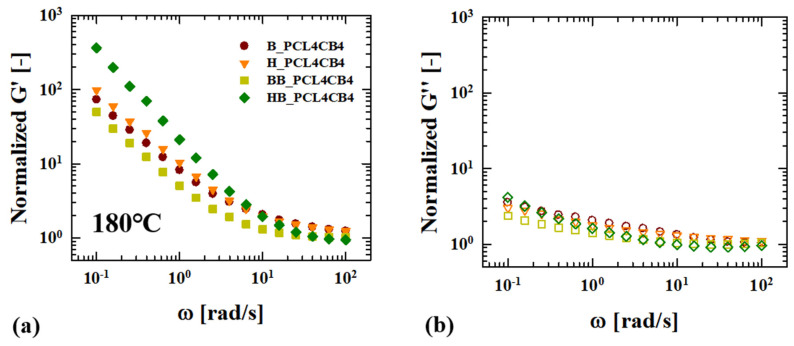
Linear viscoelasticity (G′, G″) of ternary composites depending on the mixing method at 180 °C: (**a**) The normalized storage modulus (G′) (normalized G′ = $G\prime {\left(\omega \right)}_{\mathrm{composite}}$/$G\prime {\left(\omega \right)}_{B\;\mathrm{or}\;H\_\mathrm{PLA}}$) and (**b**) normalized loss modulus (G″) (normalized G″ =$G\prime \prime {\left(\omega \right)}_{\mathrm{composite}}/G\prime \prime {\left(\omega \right)}_{B\;\mathrm{or}\;H\_\mathrm{PLA}}$).

As shown in Figure 7, if CB particles come into direct contact to form a three-dimensional percolation structure and undergo dielectric loss, CB percolation structure will be reflected in the rheological behavior of the composites as well. In order to exclude different degrees of mechanical degradation of PLA during the batch- and high- shear kneading mixing processes, and to discriminate the effect of PCL-induced CB aggregate structure on the rheological behavior of the composite clearly, G’ and G” of ternary composites are normalized with the moduli of the PLA matrix, which underwent the same mixing process with ternary composite ($\mathrm{normalized}\;G\;\prime \;\mathrm{or}\;G\prime \prime =\;\frac{G^{\prime }\left(\omega \right)\;or\;G\prime \prime \left(\omega \right)|ternary\;composites}{G^{\prime }\left(\omega \right)\;or\;G\prime \prime \left(\omega \right)|B\;or\;H\_\;PLA})$. Figure 8 shows the normalized storage (G′(ω)) and loss modulus (G″(ω)) of composites. The normalized G′ overlaps with the high-frequency region, where the matrix mainly contributes to the viscoelastic response and shows a significant increase at low frequency region, which arises from particle percolation. Nonetheless, the normalized G’ of H_PCL4CB4 or BB_PCL4CB4 shows a similar increase at the low frequency region (Figure 8a), implying that these three ternary composites have subtle differences in their percolation structure. Meanwhile, HB_PCL4CB4 clearly exhibits a pronounced increase in the normalized G′ at low frequency. Compared to the normalized G′, the normalized G″ of the ternary composite does show a slight difference depending on the mixing method (Figure 8b), meaning that the composites store energy to build up particle percolation structure. Linear viscoelasticity of a ternary composite ensures that the ternary composites with the same composition of the particles and secondary PCL phase form a different percolation structure depending on the mixing method. For the ternary composite fabricated through sequential mixing designed to produce small drops of the secondary phase before particle dispersion (HB_PCL4CB4), a pronounced increase in the normalized G′ at a low frequency occurs because the particles aggregate to form the most extended three-dimensional percolation structure compared to the other cases.

**Figure 9:**
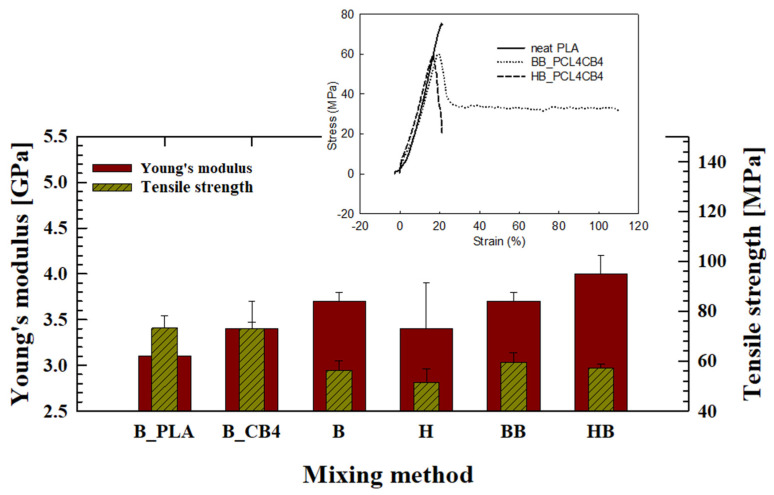
Comparison of mechanical tensile properties of ternary composites depending on mixing method.

The percolation structure change of ternary composite depending on mixing method is reflected in mechanical tensile performance, as shown in Figure 9. Mechanical tensile properties of ternary composites show different outcomes depending on mixing method even with the same composition of the particles and the secondary PCL phase. Young’s modulus of ternary composite is higher than that of PLA matrix or binary composites, and elongation at break is increased higher than 100% for BB_PCL4CB4. HB_PCL4CB4 shows the highest increase in Young’s modulus compared to the other cases. Reduction in tensile strength of ternary composites is mainly attributed to the addition of ductile PCL phase. The addition of PCL phase induces yielding behavior for ternary composites in a similar manner, while elongation at break shows different outcomes depending on mixing method. BB_PCL4CB4 shows higher elongation at break compared to HB_PCL4CB4, having a more extended percolation structure (see inset of Figure 9). It might be attributed to different degrees of mechanical degradation of PLA during the batch- and high- shear kneading mixing processes.

Electrical and mechanical property measurements show that the most extended three-dimensional percolation structure improves not only electrical properties, but also tensile properties of ternary composites. Table 5 compares the DC electrical conductivity of composites containing the same CB particles used in this study, showing that it is highly possible that the mixing method is capable of controlling particle structure to improve composite performance.

Moreover, in order to generate a percolation structure for successful CBC, the idea of an addition of a small amount of secondary phase to induce particle self-aggregation is a promising concept. It is important to disperse and distribute the secondary phase prior to the addition of particles to form particle aggregates with a high order and three-dimensional percolation structure. As the drop size of the secondary phase decreases, the secondary phase provides a large area for particle localization in a ternary system, resulting in the three-dimensional percolation of smaller aggregates and an improvement of the electrical conductivity. To further understand the particle percolation structure control, it is necessary to study the influence of chemical interaction of the secondary phase to the matrix or particle. This study provides a new concept for composite mixing and proves that mixing process has to take into account a material design step to maximize material performance.

## 4. Conclusions

The PLA/PCL/CB ternary composite exhibits a remarkable CB particle aggregation and percolation formation via PCL-induced CB self-aggregation, especially when the mixing ratio of PCL and CB was equivalent. A ternary composite shows a large variation in percolation formation depending on the mixing method, single-step or two-step mixing, leading to at least an order of magnitude increase in electrical conductivity for the system with 4 wt.% CB (from 4 × 10^−3^ to 4 × 10^−2^ S/m). Morphological, electrical, and rheological characterizations prove that CB particles form a three-dimensional percolation structure with higher connectivity with neighboring CB aggregates, through morphology control of the secondary PCL phase before the addition of particles. As the drop size of the PCL phase becomes smaller, CB particles having stronger chemical affinity to PCL than PLA have more area to localize, increasing the possibility of producing and percolating small CB aggregates. The fabrication of a conductive PLA/PCL based bio-composite through a multi-step mixing process that enhances the heterogeneity of the aggregate structure shows a high potential for many applications requiring high electrical functionalities as well as high mechanical performance unreachable by binary PLA/CB composites without PCL.

## Figures and Tables

**Table 1:** Composition and notation of the binary poly(lactic acid)/carbon black (PLA/CB) and ternary PLA/PCL (poly(caprolactone))/CB composites.

Sample Notation	Sample Composition			Ratio of PCL to CB (wt/wt)	Mixer/Mixing Condition
	Composition	PCL Based on PLA [wt.%]	CB Based on PLA [wt.%]		
B_PCLx	PLA/PCL4	4	0	-	
B_CBx	PLA/CB2	0	2	0	Internal batch mixer (B) at 180 °C, 100 rpm for 8 min
	PLA/CB3	0	3	0	
	PLA/CB4	0	4	0	
	PLA/CB5	0	5	0	
	PLA/CB6	0	6	0	
	PLA/CB8.5	0	8.5	0	
	PLA/CB13	0	13	0	
B_PCLxCBx	PLA/PCL1/CB1	1	1	1	
	PLA/PCL3/CB3	3	3	1	
	PLA/PCL4/CB4	4	4	1	
	PLA/PCL5/CB5	5	5	1	
	PLA/PCL8/CB8	8	8	1	
	PLA/PCL10/CB10	10	10	1	

**Table 2:** Notation of PLA/CB4 and (PLA/PCL4)/CB4 composites depending on mixing method.

Composite Notation	Composition	Mixer	Mixing Sequence
B_PCL4	PLA/PCL4	Internal mixer (B)	Simultaneous mixing of PLA, PCL
H_PCL4		high-shear kneading mixer (H)	Simultaneous mixing of PLA, PCL
B_CB4	PLA/CB4	Internal mixer (B)	Simultaneous mixing of PLA, CB
H_CB4		high-shear kneading mixer (H)	Simultaneous mixing of PLA, CB
B_PCL4/CB4	PLA/PCL4/CB4	Internal mixer (B)	Simultaneous mixing of PLA, PCL, CB
H_PCL4/CB4		high-shear kneading mixer (H)	Simultaneous mixing of PLA, PCL, CB
HB_PCL4/CB4		high-shear kneading mixer (H) & Internal mixer (B)	Mixing of PLA/PCL at (H), then add CB at (B)
BB_PCL4/CB4		Internal mixer (B) & Internal mixer (B)	Mixing of PLA/PCL at (B), then add CB at (B)

**Table 3:** Geometric characteristics (α_agg_, Perimeter, Aspect ratio) of CB aggregates in B_composites depending on the content of the CB and PCL phase.

Composites	α_agg._ [-]		Perimeter [nm]		Aspect ratio [-]	
	Number- Averaged	Weight- Averaged	Number- Averaged	Weight- Averaged	Number- Averaged	Weight- Averaged
B_CB3	68	120	1170	1520	1.8	1.9
B_CB4	74	150	1440	1810	1.9	2.0
B_CB5	160	300	1620	2220	1.9	2.1
B_PCL3CB3	400	780	3480	4780	2.0	2.3
B_PCL4CB4	510	1500	3980	7180	2.1	2.4
B_PCL5CB5	1100	2300	7730	13730	2.3	2.6

**Table 4:** Number-averaged and weight-averaged aggregate size (α_agg._), perimeter, and aspect ratio of CB aggregates in ternary composites depending on the mixing method.

PCL4CB4	α_agg._ [-]		Perimeter [nm]		Aspect ratio [-]	
	Number-Averaged	Weight-Averaged	Number-Averaged	Weight-Averaged	Number-Averaged	Weight-Averaged
**H**	470	850	3830	5500	1.9	2.1
**B**	510	1500	3980	7180	2.1	2.4
**BB**	640	1340	4800	7400	2.2	2.4
**HB**	1020	1770	7720	13590	2.2	2.5

**Table 5:** Comparison of DC electrical conductivity of various composites containing the CB particle used in this study.

Systems	CB Content [wt%]	2nd Polymer Content [wt%]	Mixing Method	DC Conductivity [S/m]	Ref.
**PLA/PCL/CB**	4	4	one-step	4 × 10^−3^	this study
**PLA/PCL/CB**	10	10	one-step	1	
**PLA/PCL/CB**	4	4	two-step	4 × 10^−2^	
**PLA/CB**	5	-	one-step	9 × 10^−9^	[32]
**PP/CB**	5	-	one-step	1 × 10^−9^	[33]
**HDPE/CB**	7	-	one-step	1 × 10^−10^	[34]
	10	-	one-step	1 × 10^−1^	
**PDMS/CB**	10	-	one-step	1 × 10^−3^	[35]
**PET/HDPE/CB**	5	40	one-step	1 × 10^−5^	[36]
**PP/Novolac/CB**	5	50	one-step	1 × 10^−7^	[37]
**HDPE/EVA/CB**	12	40	one-step	1 × 10^−2^	[38]
**PA6/POE-g-MA/CB**	15	40	one-step	1 × 10^−4^	[39]
